# Magnetic resonance imaging for spinocerebellar ataxia: a bibliometric analysis based on web of science

**DOI:** 10.3389/fneur.2025.1512800

**Published:** 2025-07-15

**Authors:** Zhen-Yi Liu, Lin Zhang, Zhao-Di Wang, Zi-Qiang Huang, Meng-Cheng Li, Yan Lu, Jian-Ping Hu, Qun-Lin Chen, Xin-Yuan Chen

**Affiliations:** ^1^Department of Radiology, The First Affiliated Hospital, Fujian Medical University, Fuzhou, China; ^2^Department of Rehabilitation Medicine, The First Affiliated Hospital, Fujian Medical University, Fuzhou, China; ^3^The First Affiliated Hospital of Fujian Medical University, Fuzhou, China; ^4^National Regional Medical Center, Binhai Campus of the First Affiliated Hospital, Fujian Medical University, Fuzhou, China; ^5^Clinical Center of Neuroregulation and Brain-Computer Interface, National Regional Medical Center, Binhai Campus of the First Affiliated Hospital, Fujian Medical University, Fuzhou, China

**Keywords:** spinocerebellar ataxia, magnetic resonance imaging, bibliometrics, CiteSpace, spinocerebellar ataxia type 3

## Abstract

The objective of this study was to review the history of magnetic resonance imaging (MRI) research on spinocerebellar ataxia (SCA) over the last 16 years. We conducted a comprehensive bibliometric analysis of relevant scientific literature that explores the use of MRI in studying SCA using CiteSpace. A total of 761 scientific manuscripts, published between January 2009 and March 2025 and available in the Web of Science (WoS) database, were included in this analysis. A total of 197 out of 761 articles were analyzed using CiteSpace to determine the number and centrality of publications, countries, institutions, journals, authors, cited references, and keywords related to MRI and SCA. Overall, the number of publications that use MRI to study SCA has gradually increased over the years. The United States, China, Italy, Germany, and Brazil are at the forefront in this research field; a total of 420 authors from 317 research institutions in these nations have published articles in neuroscience-related journals. Among the most cited publications are an article by Rezende et al. on brain structural damage in SCA3 patients and an review by Klockgether et al. on spinocerebellar ataxia. The keyword “spinocerebellar ataxia” has the highest frequency of occurrence. However, “feature” may become a research hotspot in the coming years based on the analysis of the keyword’s citation burst. The findings of this bibliometric study provide a summary of the last 16 years of SCA research using MRI technology. More importantly, the present study identifies current trends and future research hotspots in the field, helping researchers to identify new and unexplored research areas.

## 1 Introduction

Spinocerebellar ataxias (SCAs) are a genetically heterogeneous group of autosomal dominantly inherited progressive neurodegenerative disorders (1). They are relatively rare and primarily affect adults, with a global estimated prevalence of <5.6 cases per 100,000 people, and an average of 3 cases per 100,000. Currently, more than 40 subtypes are known (2), with is spinocerebellar ataxia type 3 (SCA3) being the most common (3). The core symptoms of SCAs include gait ataxia, oculomotor disturbances, and dysarthria (4, 5). Currently, patients with SCA can be diagnosed through characteristic genetic testing (6).

Consistent with other neurodegenerative diseases, the clinical evaluation scale score is the most widely used outcome measure in clinical studies of SCA. Ataxia scales were mainly developed to quantify the impact of a known disease on each patient, enabling comparisons with other patients who have the same disorder (7). Among these scales, the Scale for the Assessment and Rating of Ataxia (SARA) and the Cooperative Ataxia Rating Scale (ICARS) are currently the most widely used and effective indices because of their sensitivity to changes and effortless operation (8). However, both SARA and ICARS have certain limitations. They have floor and ceiling effects, making them not very effective when the symptoms are either very mild or very severe. Above all, these scoring scales are inherently subjective (9) and often need to be supplemented with more reliable evaluation indicators.

At present, the use of magnetic resonance imaging (MRI) technology to explore the pathogenesis and potential biomarkers of SCA is gaining increasing attention (10). Structural or functional abnormalities of the brain tissue can be observed by different MRI techniques, including morphometric magnetic resonance imaging (MMRI), diffusion tensor imaging (DTI), blood oxygen level-dependent functional MRI (BOLD fMRI), and magnetic resonance spectroscopy (MRS) (11). Many related studies have revealed that there is a strong correlation between the MRI and scale results for SCA diagnosis and follow-up (12–14). However, there is a lack of summary and evaluation of the literature characteristics, research directions, and research hotspots related to the use of MRI in clinical cases of SCAs.

Bibliometrics is a quantitative statistical analysis tool that measures the influence and impact of research articles (15), and it is largely dependent on visualizing processing tools such as CiteSpace (16, 17). CiteSpace is a software package known to be a visual analytical tool for identifying the landscape, pattern, and emerging trends in a field of research or any knowledge domain based on selected literature databases, such as Web of Science (WoS) and Scopus (18, 19).

In this study, we conducted a bibliometric analysis of scientific articles published between 2009 and 2025 that focus on the use of MRI to study SCA. This analysis aims to outline the current research landscape, future research trends, and hotspots in this field.

## 2 Materials and methods

### 2.1 Data acquisition and search strategy

The data for bibliometric analysis was obtained from the WoS Core Collection (WoSCC), which includes large-scale, multidisciplinary, high-impact, international, and comprehensive academic journals (20). The following search terms were used to gather relevant literature from the WoSCC (Table 1).

**Table 1:** Search strategy from the WoS core collection.

Set	Results
#3	#1 AND #2
#2	(((((((((((((TS = (MRI)) OR TS = (“magnetic resonance imaging”)) OR TS = (“mr imagimg”)) OR TS = (DTI)) OR TS = (radiology)) OR TS = (neuroimaging))) OR TS = (fMRI)) OR TS = (sMRI)) OR TS = (“functional magnetic resonance imaging”))) OR TS = (structural magnetic resonance imagimg)) OR TS = (“diffusion tensor imaging”)) OR TS = (3D-T1)
#1	(((((((((((((((((((((((((((((((((((((TS = (“spinocerebellar ataxia”)) OR TS = (“Machado-Joseph disease”)) OR TS = (SCA)) OR TS = (MJD))OR TS = (SCA1)) OR TS = (SCA2)) OR TS = (SCA3)) OR TS = (SCA4)) OR TS = (SCA18)) OR TS = (SCA25))) OR TS = (SCA38)) OR TS = (SCA43)) OR TS = (SCA46)) OR TS = (SCA7))) OR TS = (SCA8)) OR TS = (SCA10)) OR TS = (SCA14))) OR TS = (SCA15)) OR TS = (SCA17)) OR TS = (SCA35)) OR TS = (SCA40)) OR TS = (SCA43)) OR TS = (SCA20)) OR TS = (SCA19)) OR TS = (SCA22)) OR TS = (SCA21)) OR TS = (SCA12)) OR TS = (SCA27)) OR TS = (DRPLA)) OR TS = (SCA28)) OR TS = (SCA36)) OR TS = (SCA34)) OR TS = (ATN1)) OR TS = (DNMT1))

The inclusion criteria included online articles and review articles published from 1 January 2009 to 1 March 2025. Only research articles published in English was considered. Two independent investigators performed the article screening process. A third investigator was involved in the decision of accepting or rejecting a manuscript for further analysis in case of discrepancies.

This study adhered to the guidelines outlined in “How to conduct a bibliometric analysis: an overview and guidelines” (51).

### 2.2 Data analysis

We exported the retrieved articles in plain text format to CiteSpace 6.1. R6 and VOSviewer version 1.6.19 for further analysis. Our performance analysis included the following publication- and citation- related indexes such as publication-related metrics, citation-related metrics, and citation- and publication-related metrics. Bibliometric visualization was also carried out using the main procedural steps in CiteSpace 6.1. R6, including time slicing, thresholding, modeling, pruning, merging, and mapping (21). Nodes in different maps represent authors, institutions, countries, or keywords. The size of a node represents the frequency of occurrence of a citation, while the color of a node represents its year of publication. The purple edges of nodes reflect the centrality of the corresponding nodes, and these nodes with high centrality are usually recognized as hotspots or turning points in the field (22). We use VOSviewer version 1.6.19 for keyword analysis and obtain the Density view map. Each point in the map has a color that depends on the number of items near that point and the importance of adjacent items (23).

## 3 Results

### 3.1 Overview of publication numbers for different years, countries, and scientific institutions

**Figure 1:**
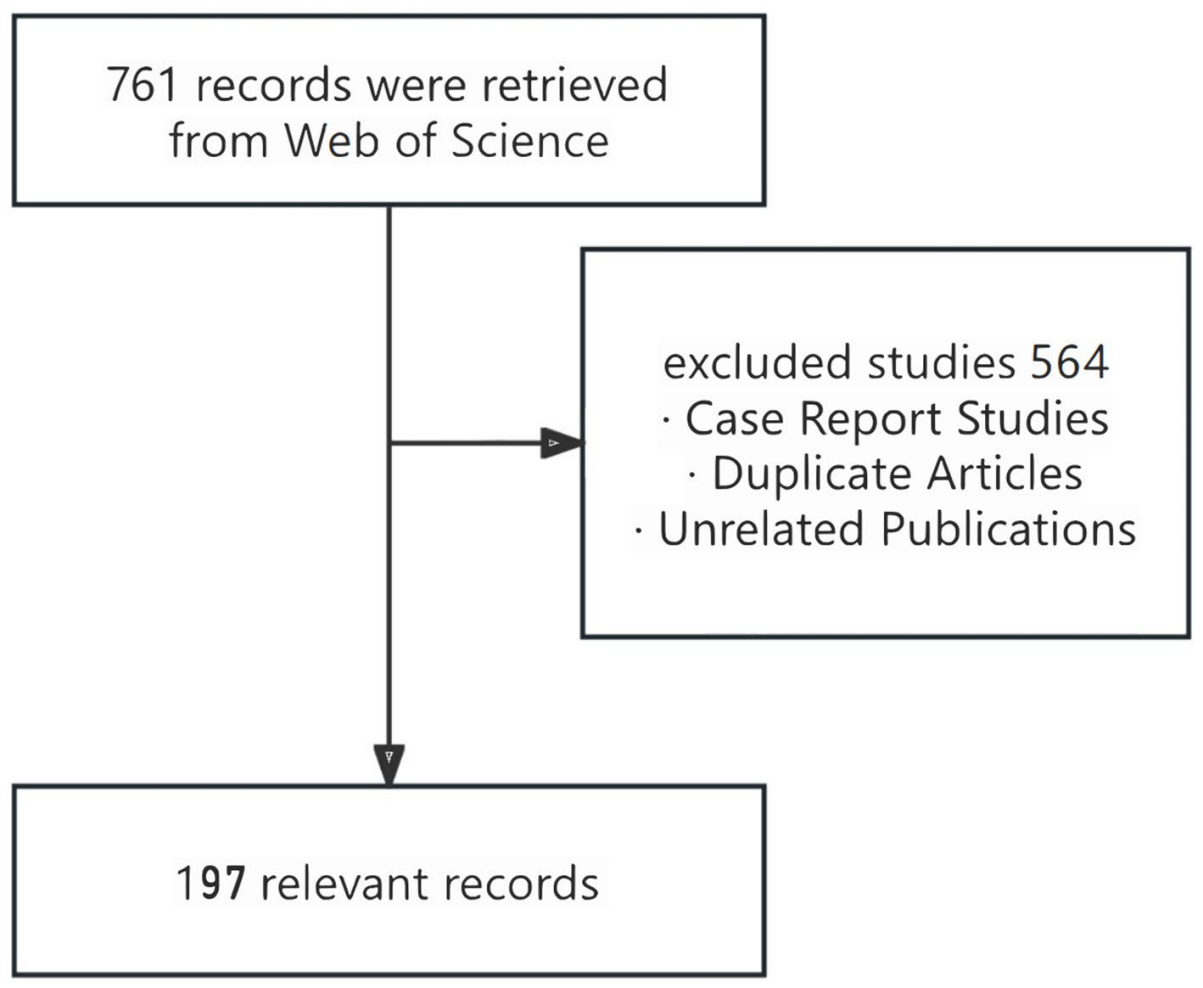
Database search flow chart (software: WPS Office 2023, Kingsoft, China).

A total of 761 scientific publications met the inclusion criteria. After excluding case report studies, duplicate articles, and unrelated publications that escape our filtering conditions, a final sample of 197 studies were found suitable and was used in the bibliometric analysis (Figure 1).

**Figure 2:**
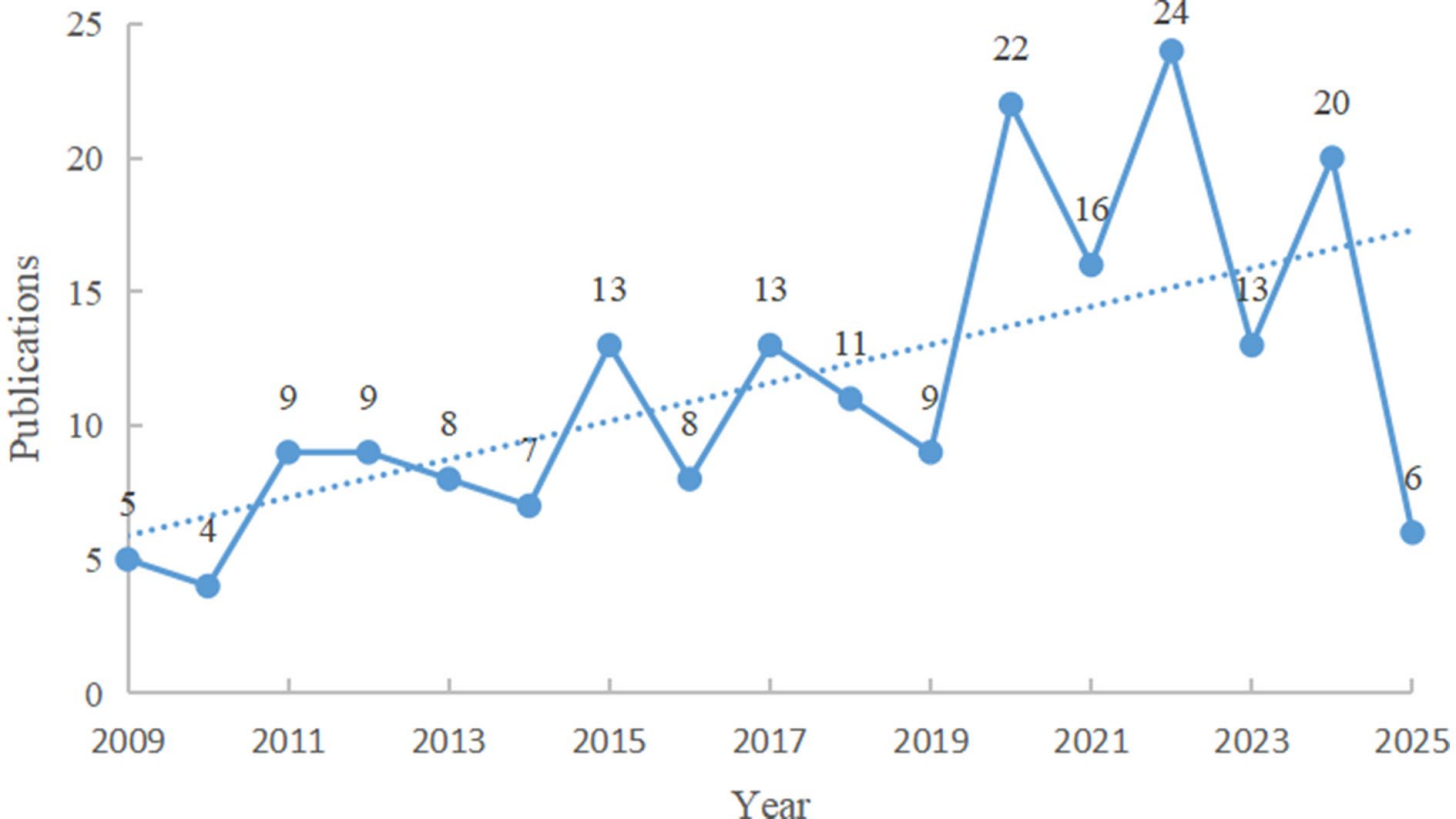
Annual number of publications about MRI for the study of SCA. The vertical y-axis represents the number of publications, while the horizontal x-axis represents the publication year. The numbers on the plot correspond to the number of publications per year. The dashed line indicates the publication trend. The graph was obtained using Office 2016 and the data from Citespace6.1.R2 analysis.

Although the number of MRI and SCA-related publications fluctuated during our study period, the overall trend rose, with a particularly rapid growth starting in 2020 (Figure 2). Only six 2025 publications were included in the analysis. This low number is probably due to a reduced sampling time, limited to the first 2 months of that year.

**Figure 3:**
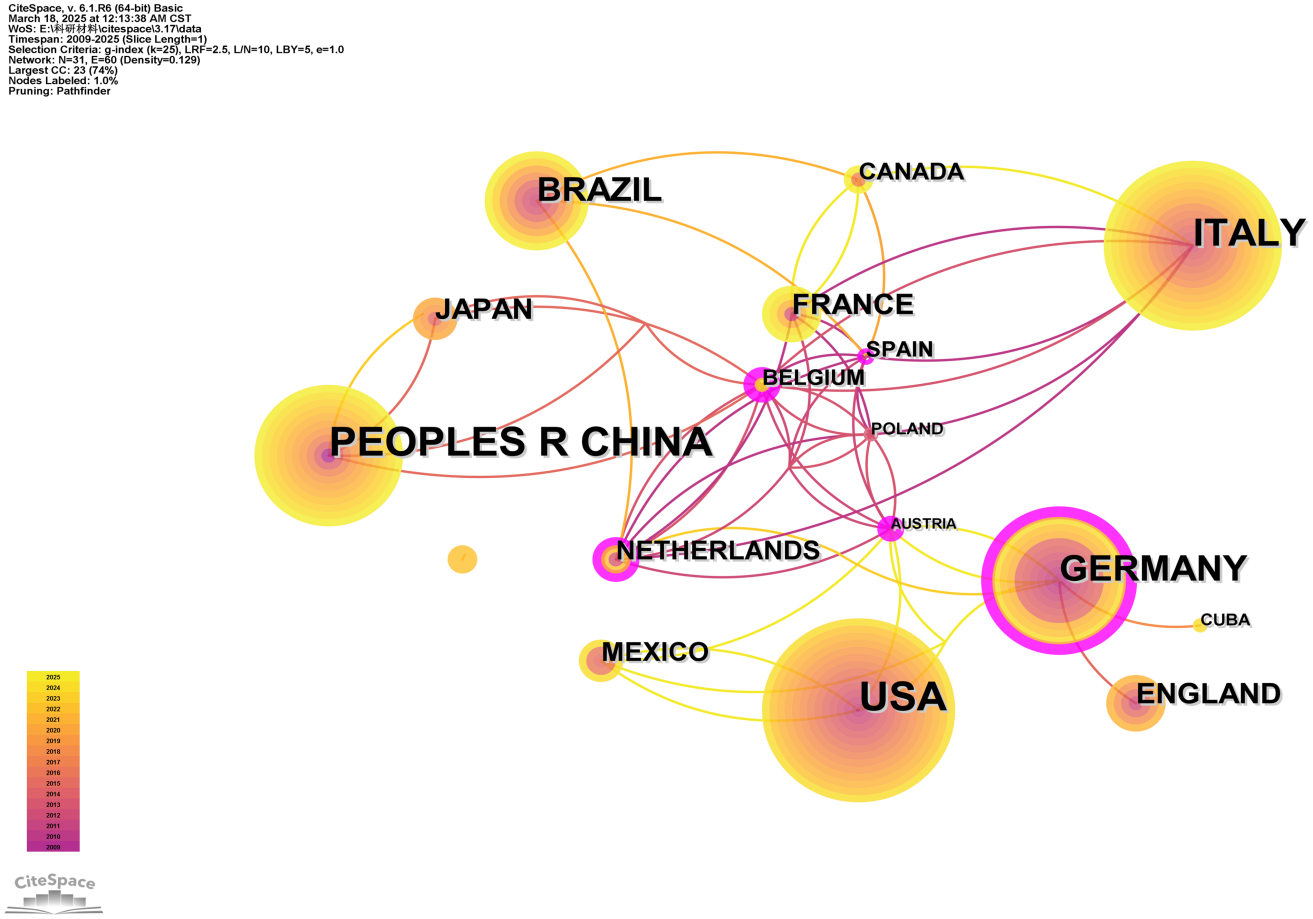
Country collaborative network analysis. The thickness of the outermost purple ring represents the size of the centrality. Other than that, different colors represent different years. The lines represent the connections between the nodes.

A total of 43 countries have published articles on the use of MRI in SCA cases, and Table 2 shows the 10 countries with the highest numbers, with the top five countries covering over 90% of the publications. These countries, including China (54), the United States (United States) (45), Italy (34), Germany (27), and Brazil (23), are expressed in country collaborative network analysis as the size of the node (Figure 3). Surprisingly, we found that while the total number of MRI and SCA-related publications from China is the highest, Austria has the highest centrality (BC = 0.46), which is expressed in country collaborative network analysis as the thickness of the outer purple ring. Furthermore, we observed that the United States, Italy, and Brazil have a high volume of publications, but their centrality is low.

**Table 2:** Top 10 countries by publications.

Rank	Count	BC	Year	Country
1	54	0.10	2009	PEOPLES R CHINA
2	45	0.10	2009	UNITED STATES
3	34	0.04	2010	ITALY
4	27	0.20	2010	GERMANY
5	23	0.00	2009	BRAZIL
6	15	0.00	2009	JAPAN
7	13	0.01	2010	FRANCE
7	13	0.00	2010	ENGLAND
9	12	0.00	2011	MEXICO
10	10	0.13	2010	NETHERLANDS

Count is the number of the publication; BC value represents the degree of centrality = the number of relational ties an article or a research component (e.g., authors, countries, institutions, journals) has in the network.

**Figure 4:**
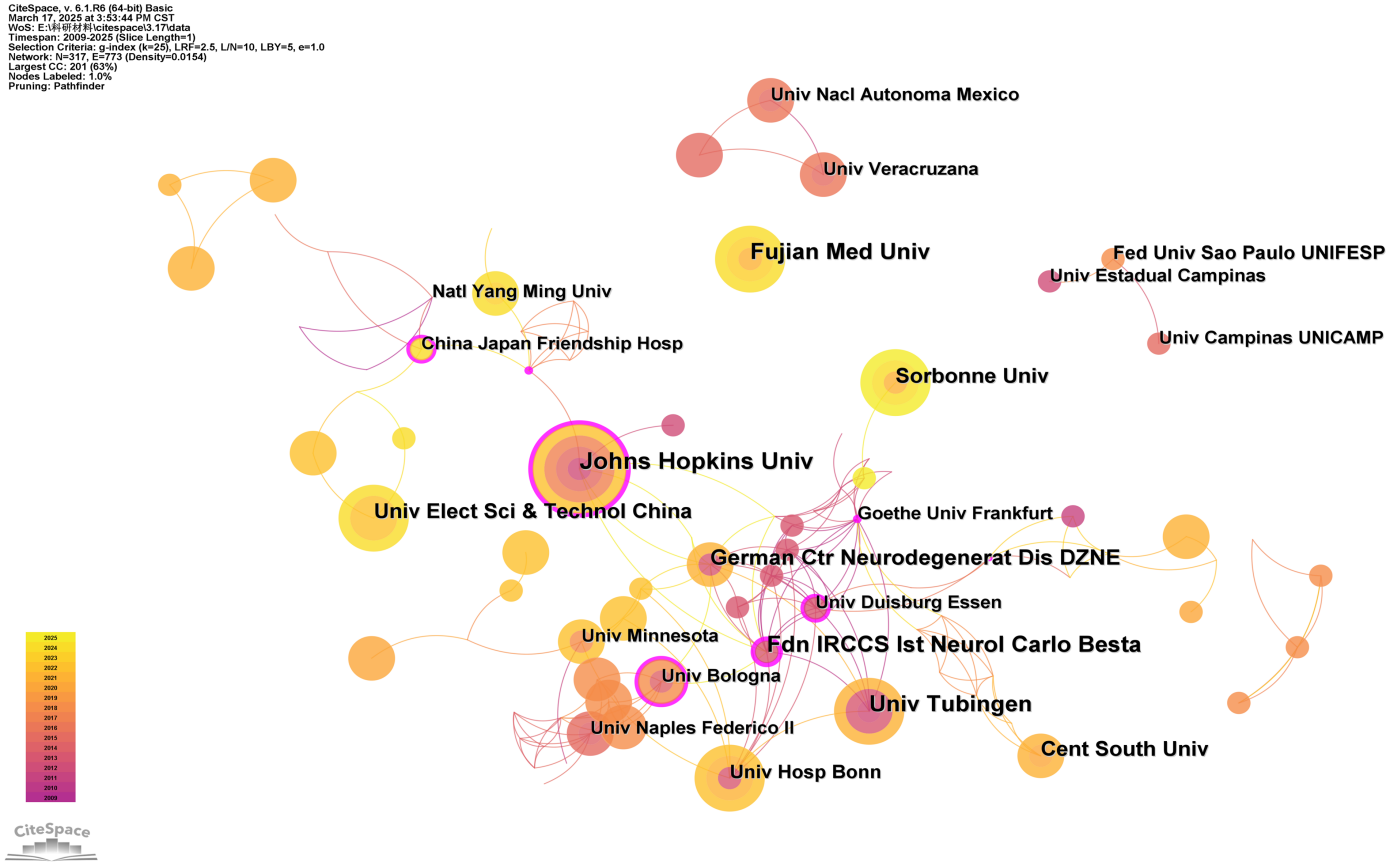
Institution collaborative network analysis. The thickness of the outermost purple ring represents the size of the centrality. Other than that, different colors represent different years. The lines represent the connections between the nodes.

A total of 317 institutions have conducted research on SCAs using MRI, most of which are located in the United States, China, and Germany. The institution with the highest number of publications is Johns Hopkins University in the United States, with 10 publications; this result is expressed in the institution’s collaborative network analysis as the node size. Furthermore, the core institution remains Johns Hopkins University. This result is expressed in the institutional collaborative network analysis as the thickness of the outer purple ring (BC = 0.31) (Figure 4).

### 3.2 Overview of the most cited journals, publications, and authors

Overall, the top-ranked journals are all classic journals in the relevant fields of neuroscience. All the journals ranked within the most cited top 10 accumulate more than 89 citations (Table 3). The journal with the highest co-citation frequency is BRAIN, with 165 citations, closely followed by the other four journals in the top 5, each of which is cited more than 140 times. Most of the top 10 cited journals are owned by publishing groups in the United States and the United Kingdom (UK). Except for Archives of Neurology and Cerebellum, all journals are among the highest-ranked journals in their category (Q1 quartile) as reported by Clarivate Journal Citation Reports.

**Table 3:** Top 10 journals ranked by co-cited frequency of publications.

Rank	Journal	Co-cited frequency	BC	Country	Impact factor (2022)	Quartile in category (JCR)	Year
1	Brain	165	0.00	United Kingdom	14.5	Q1	2009
2	Neurology	159	0.00	United States	9.9	Q1	2009
3	Movement Disorders	157	0.00	United States	8.6	Q1	2009
4	NeuroImage	153	0.01	United States	5.7	Q1	2009
5	Cerebellum	149	0.00	United States	3.5	Q3	2009
6	Journal of Neurology	137	0.01	Germany	6.0	Q1	2009
7	Annals of Neurology	121	0.01	United States	11.2	Q1	2009
8	Lancet Neurology	95	0.01	United Kingdom	48	Q1	2009
9	Journal of Neurology, Neurosurgery and Psychiatry	91	0.03	United Kingdom	11.0	Q1	2009
10	Archives of Neurology	89	0.01	United States	/	/	2010

**Figure 5:**
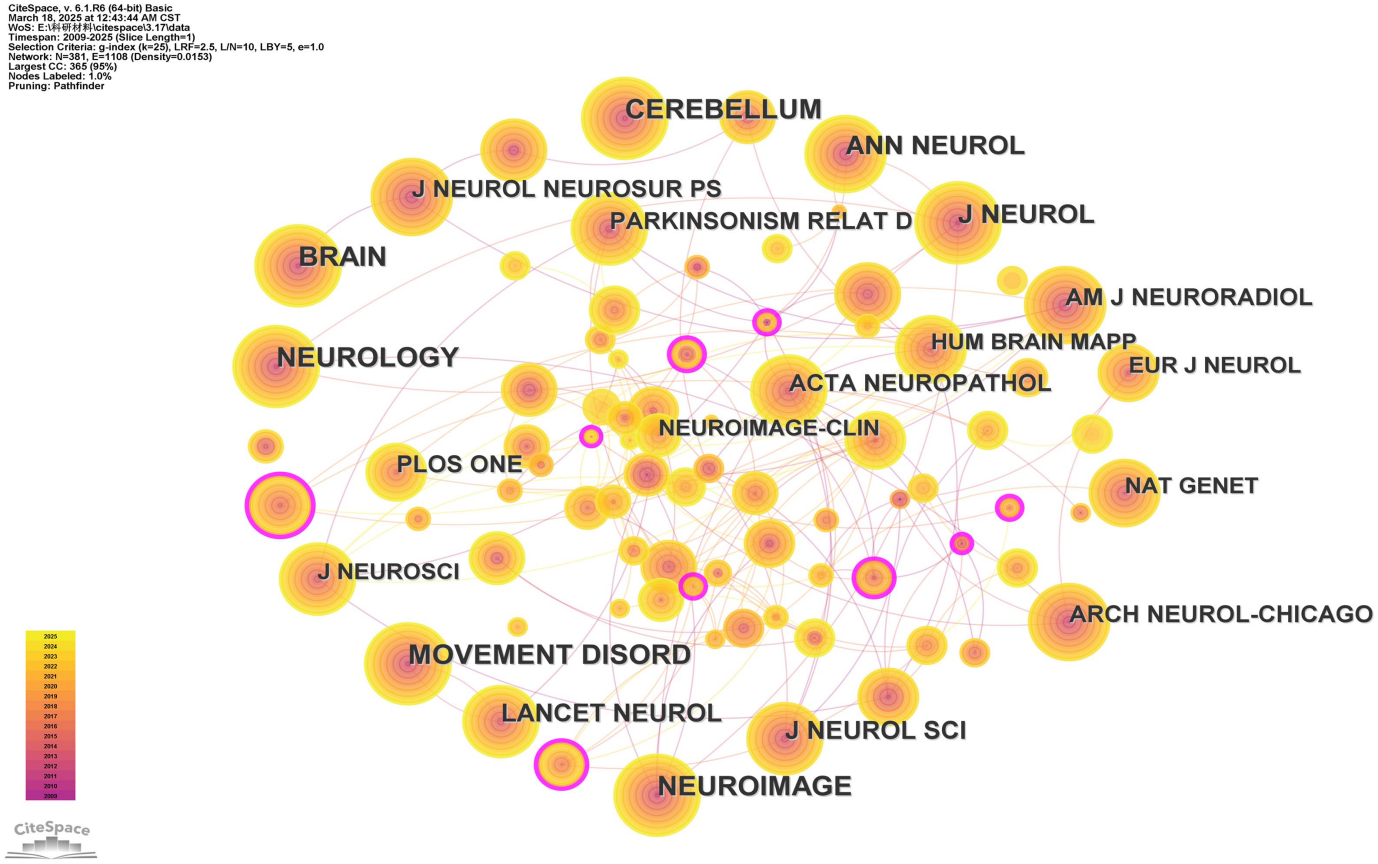
Cited journals network analysis. The thickness of the outermost purple ring represents the size of the centrality. Other than that, different colors represent different years. The lines represent the connections between the nodes.

Unexpectedly, the 10 journals with the most citations are not among those with the highest BC (between centrality) values, less than 0.05. The journal with the highest between-centrality is Brain Research (BC = 0.16), which showed up as the thickest purple ring in the Cited Journals network analysis (Figure 5).

Of the 197 articles included in the analysis, only one among the top 10 most cited publications is a review article. The review from Klockgether et al. (1) in Nature Reviews Disease Primers, has the highest impact factor (79.0) among all the publications in this project, and it is hold the highest citation record (29 citations), which is same to a manuscript published in Annals of Neurology (impact factor 8.1) and that details the “Structural signature of SCA3: from presymptomatic to late disease stages” (24) Indeed, most scientific articles using MRI in the study of SCA focus on exploring the changes of brain microstructure in SCA patients and using structural biomarkers to optimize the diagnosis, prediction, and treatment of diseases (Table 4).

**Table 4:** Top 8 publications ranked by co-cited frequency.

Rank	Title	Reference type	Co-cited frequency	BC	Journal	Impact factor (2023)	JCR quartile	Year
1	Spinocerebellar ataxia	Review	29	0.03	Nature Reviews Disease Primers	79.0	Q1	2019
2	Structural signature of SCA3: From presymptomatic to late disease stages	Clinical trial	29	0.02	Annals of Neurology	8.1	Q1	2018
3	Visualization, quantification, and correlation of brain atrophy with clinical symptoms in spinocerebellar ataxia types 1, 3, and 6	Clinical trial	18	0.06	Annals of Neurology	8.1	Q1	2010
4	Autosomal dominant cerebellar ataxias: Imaging biomarkers with high effect sizes	Clinical trial	17	0.12	NeuroImage-Clinical	3.4	Q2	2018
5	Gray matter atrophy patterns within the cerebellum-neostriatum-cortical network in SCA3	Clinical trial	15	0.06	Neurology	8.4	Q1	2020
6	Characterization of Lifestyle in Spinocerebellar Ataxia type 3 and Association with Disease Severity	Clinical trial	13	0.02	Movement Disorders	7.4	Q1	2021
7	A multimodal evaluation of microstructural white matter damage in spinocerebellar ataxia type 3	Clinical trial	13	0.07	Movement Disorders	7.4	Q1	2013
8	Genotype-specific patterns of atrophy progression are more sensitive than clinical decline in SCA1, SCA3 and SCA6	Clinical trial	13	0.01	Brain	11.9	Q1	2013

### 3.3 Analysis of authors and cited authors

**Figure 6:**
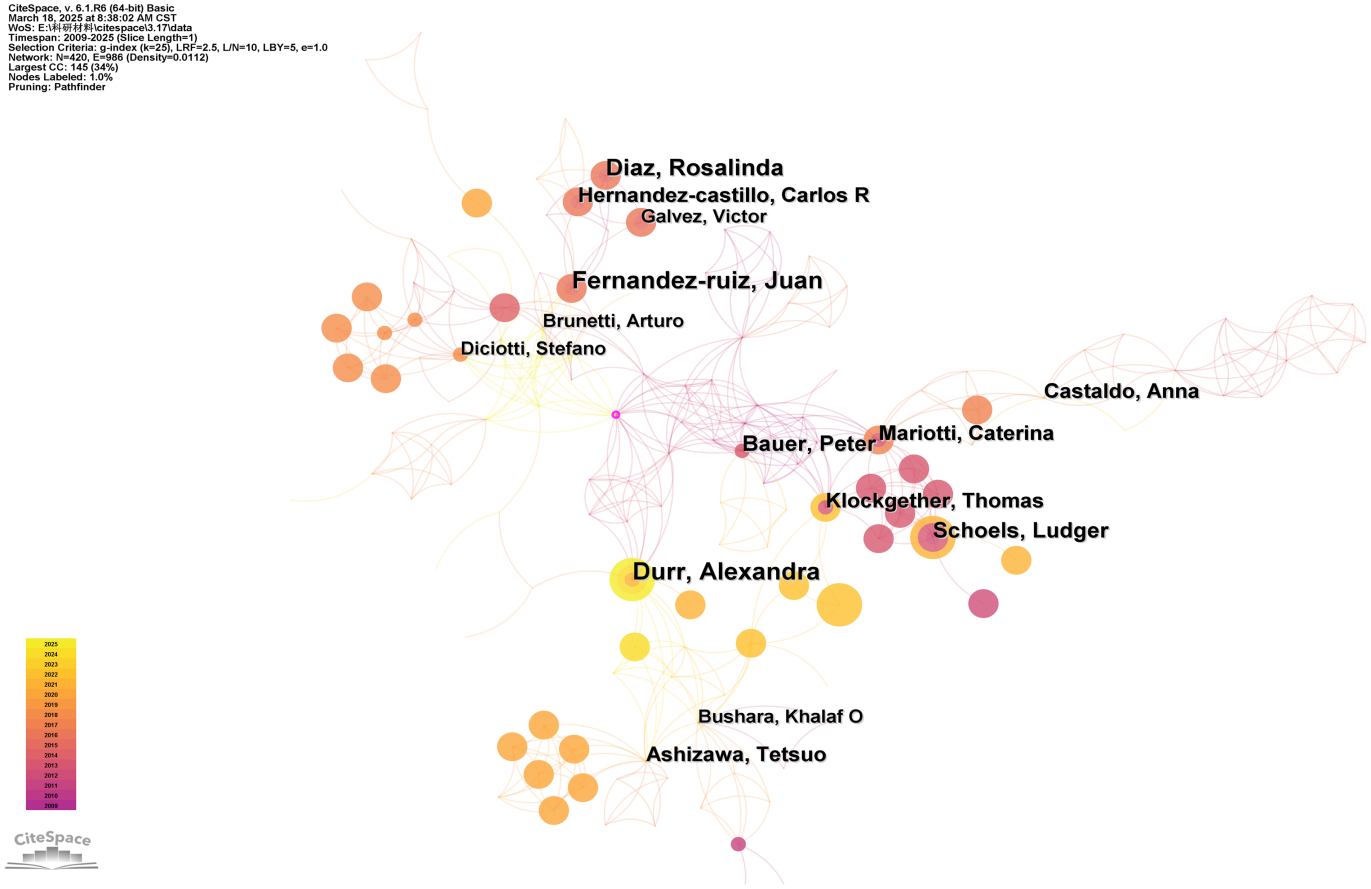
Network analysis of authors. The thickness of the outermost purple ring represents the size of the centrality. Other than that, different colors represent different years. The lines represent the connections between the nodes.

The collaborative author network showcases author’s productivity and collaborations. A total of 420 scholars have used MRI to research SCAs (Figure 6). The authors with the highest publication volume (8 articles) are Dr. Juan Fernandez-Ruiz (Universidad Nacional Autónoma de México, Mexico) and Dr. Alexandra Durr (Sorbonne University, France). Regarding centrality, the BC values of the two authors were 0.06 and 0.02, respectively. The author with the highest centrality is Dr. Sylvia Boesch (Medical University of Innsbruck, Austria) (BC = 0.10), who has published a total of 3 articles.

**Figure 7:**
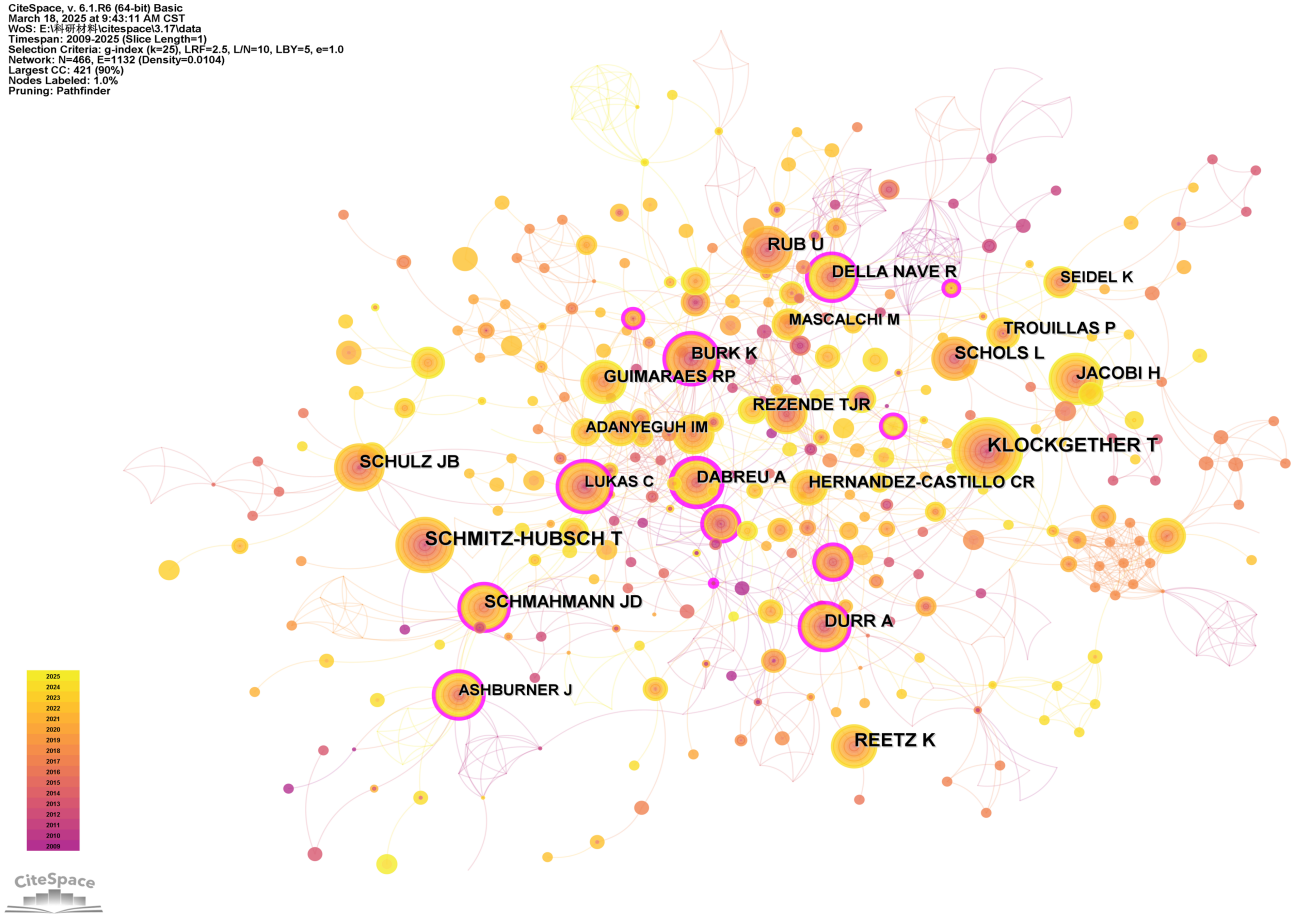
Network analysis of cited authors. The thickness of the outermost purple ring represents the size of the centrality. Other than that, different colors represent different years. The lines represent the connections between the nodes.

Dr. Klockether is the most cited author (80 citations) in the narrow field of MRI usage for SCA research (Figure 7), and Dr. Anelyssa D’Abreu (University of Campinas, Brazil) has the highest centrality in the citations network (BC = 0.23).

### 3.4 Analysis of keywords

**Figure 8:**
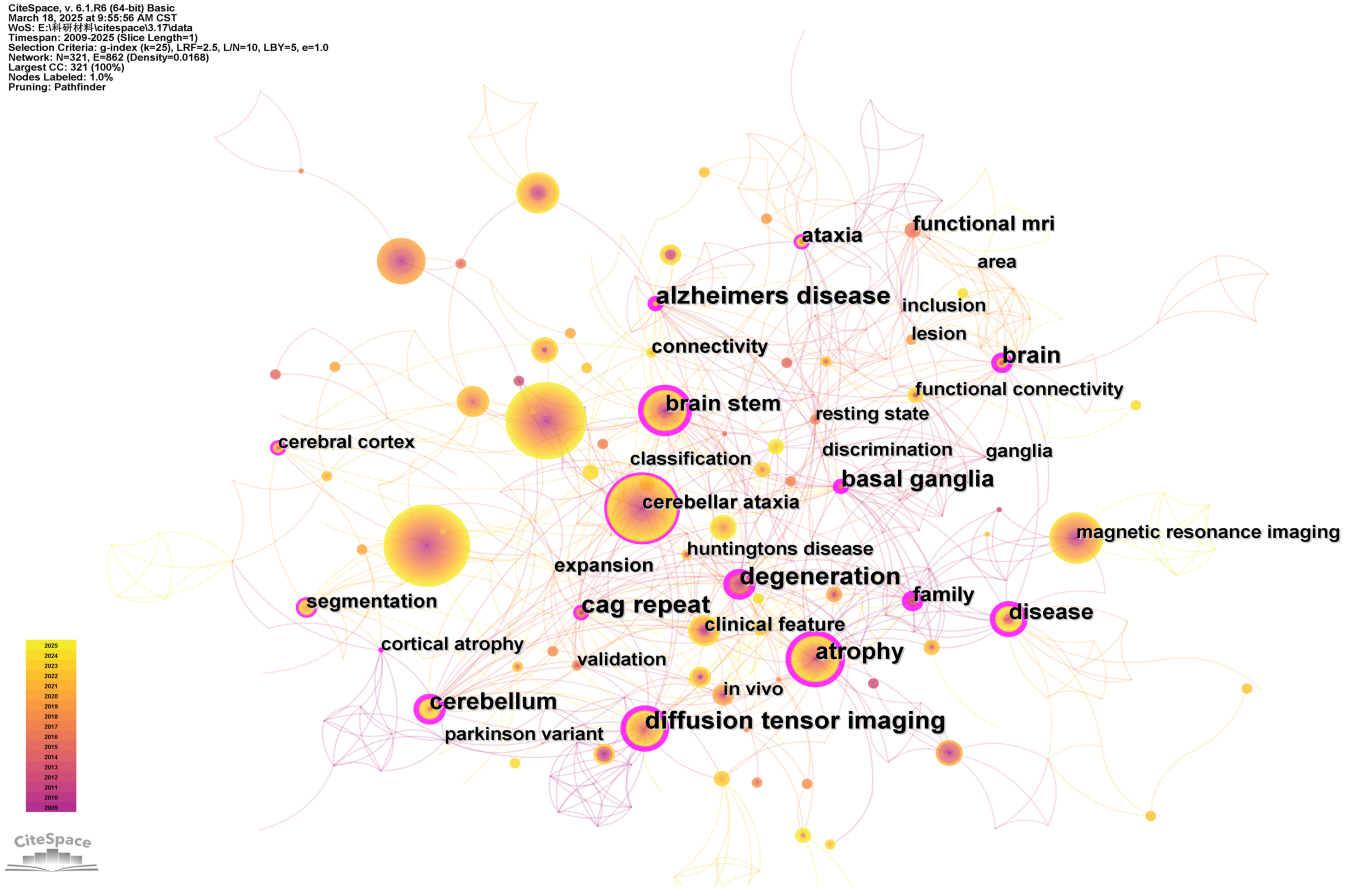
Analysis of keyword occurrence.

Of the 327 keywords we collected, 212 only appeared once, accounting for 64.8%. Figure 8 shows that the keyword “spinocerebellar ataxia type 3” is the most popular keyword with 62 occurrences. The most central keyword is “diffusion tensor imaging” (BC = 0.38), followed closely by “alzheimers disease” (BC = 0.34) and “basal ganglia” (BC = 0.29) (Table 5).

**Figure 9:**
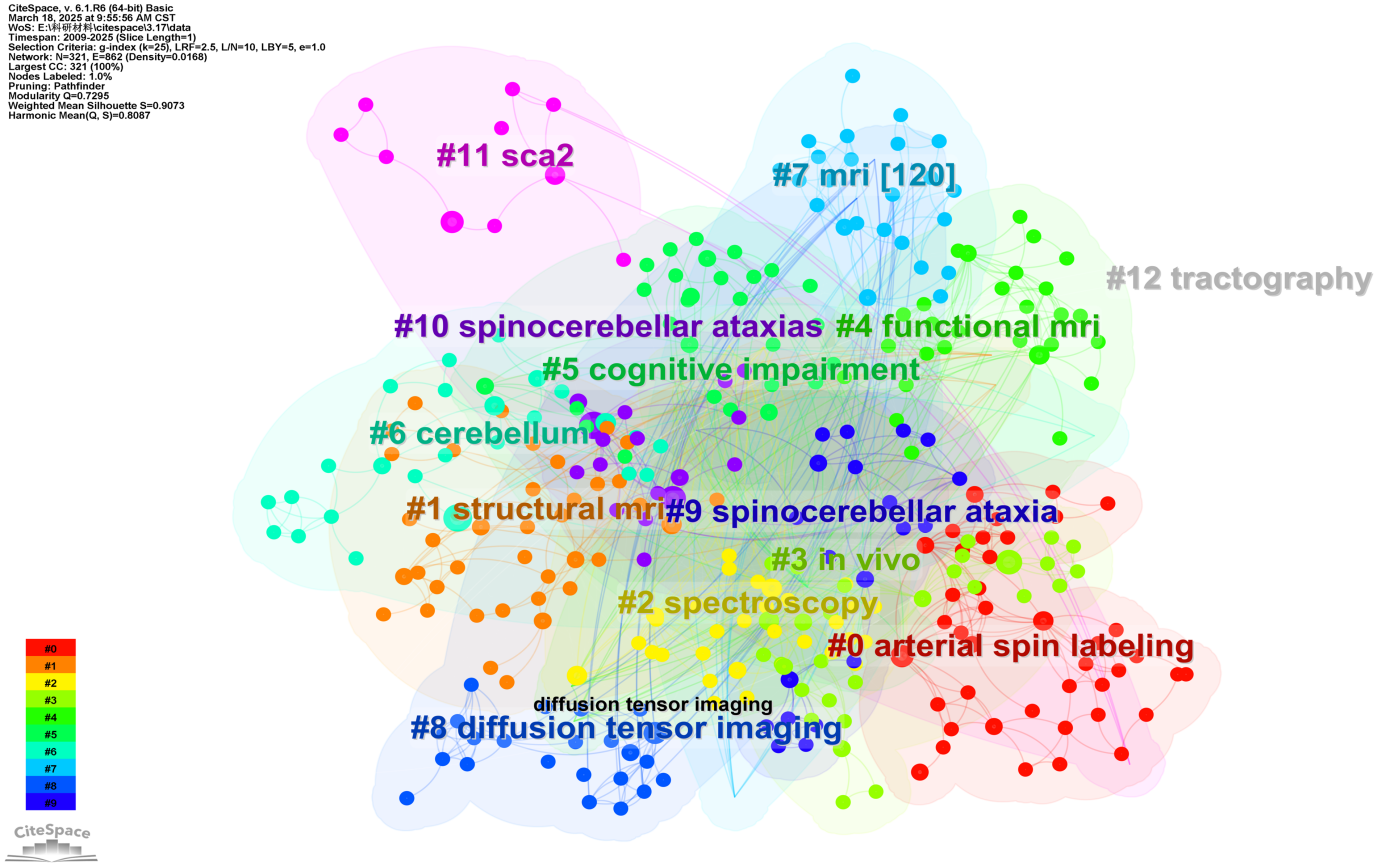
Keyword clustering mapping. Different colors represent different clusters, and cluster names are the most representative keywords in the cluster. The smaller the number in front, the more keywords are included in the cluster.

By using the Log-Likelihood Ratio (LLR) to assess the significance of associations between the keywords (nodes) in the keyword network (Figure 8), we observed that the research involving MRI for the study of SCA can be divided into 13 clusters (Figure 9). LLR (Log-Likelihood Ratio) provides unique labels to the clusters with adequate coverage of core literature compared to other labeling extraction algorithms (25). The top three clusters are: (i) arterial spin labeling (41), (ii) structural MRI (39), and (iii) spectroscopy (31). This represents that these three clusters contain the most keywords. Cluster names are the most representative keywords among them.

**Table 5:** Top 10 keywords co-occurrence frequency.

Number of occurrences	BC	Year of occurrence	Keywords
62	0.01	2009	Spinocerebellar ataxia type 3
56	0.03	2010	Spinocerebellar ataxia
45	0.07	2009	Magnetic resonance imaging
44	0.11	2009	Cerebellar ataxia
37	0.25	2009	Atrophy
33	0.38	2009	Diffusion tensor imaging
26	0.04	2010	Voxel based morphometry
24	0.09	2009	Clinical feature
21	0.02	2011	Damage
21	0.21	2009	Brain stem

Year of occurrence refers to the year in which it first appeared.

**Figure 10:**
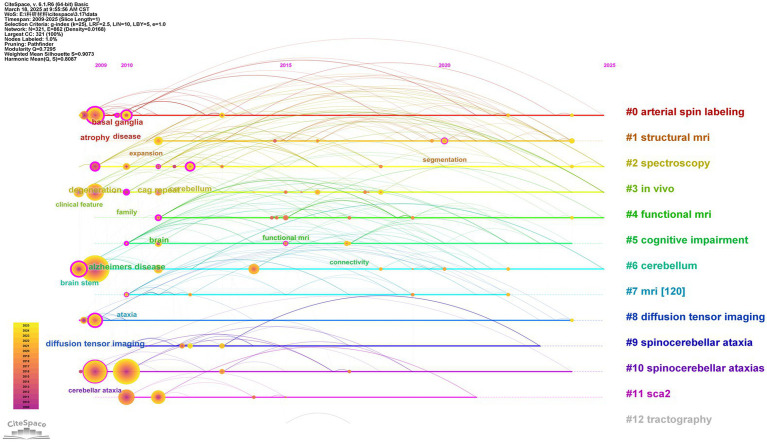
Keyword timeline view. The labels and IDs of the cluster are on the right side of the figure. The position of the node on the timeline represents the year in which the keyword first appeared.

We performed a keywords timeline analysis to display the evolution of the 327 keywords (Figure 10). Keyword cluster analysis can reveal the evolution and trends of keywords within each cluster. The keywords “atrophy,” “degeneration,” “diffusion tensor imaging,” “brain stem,” and “basal ganglia” related to the SCA research via MRI emerged in 2009 and have remained a research hotspot to date. In addition, the keyword “voxel-based methodology” first appeared in 2010 and remained a research hotspot until 2020. Between 2015 and 2017, and again in 2022, the keyword “functional MRI” was relatively popular before regaining attention between 2020 and 2024.

**Figure 11:**
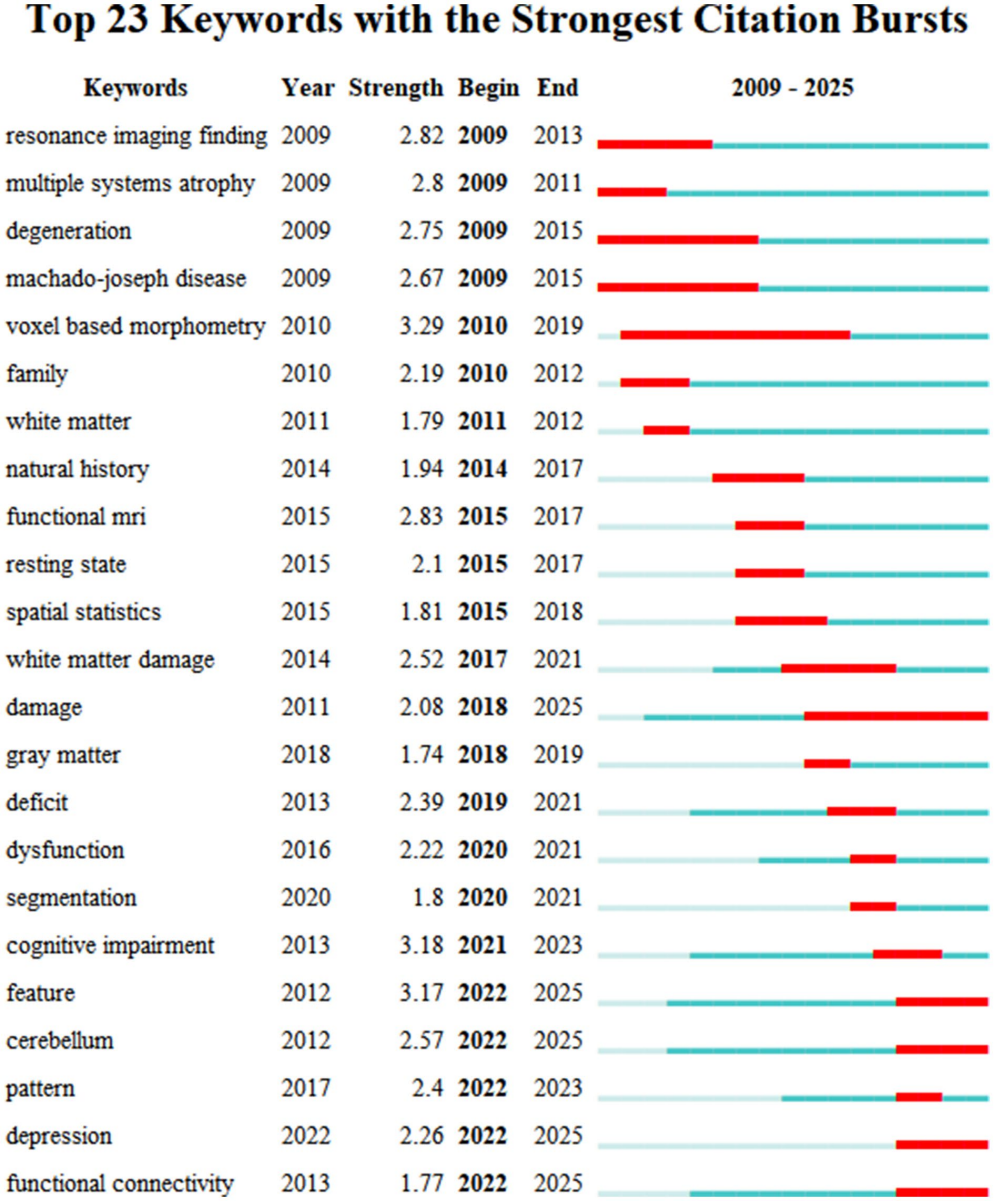
Analysis of the keyword’s citation burst. Year indicates the year of the first occurrence. Strength indicates the magnitude of the burst. Begin and end show the year span in which the burst happened. The blue line on the graph represents the overall time interval, while the red line specifically marks the time period during which a keyword exhibited a burst.

Furthermore, the 23 keywords with the strongest citation bursts were identified (Figure 11). Bursts reflect the emergence of a keyword during a specific period (18). These burst keywords were detected based on the increase in the frequency in the publications in that year, regardless of the total usage (26). The most pronounced and influential burst was associated with keywords “voxel-based morphometry” (Strength = 3.29) and “cognitive impairment” (Strength = 3.18). Four keywords, “feature,” “cerebellum,” “depression,” and “functional connectivity,” have experienced a burst in recent years, suggesting that they may become prominent research topics in the coming years.

**Figure 12:**
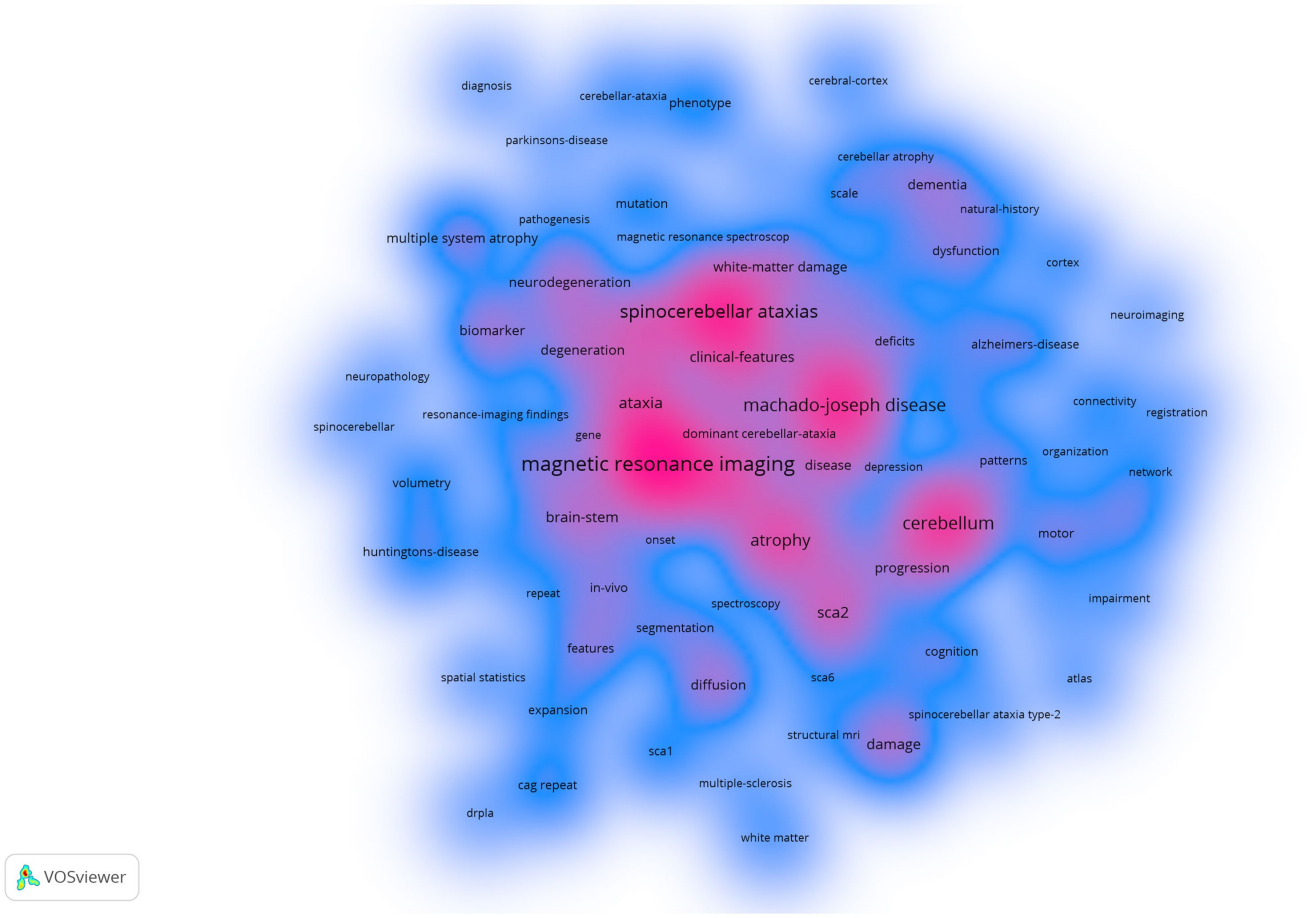
Screenshot of the density view.

Furthermore, we employed the VOSviewer software for keyword analysis (Figure 12). Density views are especially valuable for comprehending the overarching structure of the map and highlighting its most critical regions. The significance of terms such as “Machado-Joseph disease,” “atrophy,” and “voxel-based methodology “within this research domain is more distinctly demonstrated.

## 4 Discussion

To the best of our knowledge, this research marks the first instance in which we have used CiteSpace software to conduct a bibliometrics analysis of original research articles and reviews related to the use of MRI in SCA. This analysis spans from January 2009 to March 2023, utilizing data from the Web of Science database. Bibliometric analyses have become a powerful tool for summarizing the current state of knowledge of a certain research topic (27) as they present visual maps to display the current research overview more intuitively, and, to some extent, indicate future development trends. After excluding studies that did not meet the inclusion criteria, we selected 197 papers with 420 authors from 317 institutions in 44 countries for analysis. We used CiteSpace software to perform a comprehensive analysis of countries, research institutions, journals, authors, publications, and keywords to summarize the research overview of SCAs related to MR technologies and predict potential research hotspots in this field.

We observed that the number of publications exhibits an increasing trend of volatility over time (Figure 2). This indicates that the application of MR technology in SCA research is growing and becoming increasingly widespread. Several factors contribute to this trend: (1) Despite being a group of rare diseases, the increasing global population has made rare diseases, including SCAs, a public health priority, necessitating focused research attention (28); (2) Advances in MRI technology have expanded non-invasive detection possibilities, enabling more comprehensive evaluation of the microstructure and function of the brain in SCA patients; (3) Understanding the specific characteristics of SCAs, such as neuroradiology, can provide valuable auxiliary information in the diagnostic process (29). The annual growth of publications related to the application of MRI in SCAs is attributable to the outstanding contributions made by countries, institutions, journals, and researchers in this field.

We observed that research on this rare disease is gradually deepening in countries such as the United States, China, Italy, Germany, and Brazil (Figure 3). Notably, the United States and Germany have emerged as significant contributors to this research field, with their publication volumes and centrality ranking among the highest globally. This finding reflects the European and American efforts at establishing large-scale SCA cohorts within their borders. The accomplishment is attributed to sufficient funding, robust research institutions, and outstanding researchers, placing them at the forefront of international research. In developing countries like China and Brazil, research efforts are notably focused on Fujian Medical University, Central South University, and Campinas State University. The focus of these two countries in this field may be linked to their large populations (30). Notably, China and Brazil exhibit lower centrality than their European counterparts. This observation may be attributed to the relatively recent development in the biopharmaceutical field, given their status as developing countries (31). Furthermore, institutions and authors in Brazil and China currently lack international communication. Possible factors contributing to this phenomenon encompass linguistic and cultural disparities, divergences in research methodologies within academic systems, restricted avenues for information acquisition and dissemination, and the absence of collaborative mechanisms and platforms. Comprehending these potential causes can facilitate the identification of strategies to strengthen bilateral relations. Due to the lack of multivariate cooperation, the MRI equipment and techniques commonly used in different countries may differ. Therefore, the technology used in the United States, Germany, China, Italy, Brazil, and other countries will dominate the analysis. To foster future research, increased collaboration with scholars from the United States and Europe is essential. Multilateral cooperation via global and regional networks, including the GlobalSCA, EuroSCA, and the Pan-American Genetic Ataxia Network, is essential for advancing and expanding this research.

It is important to highlight that the 10 most cited journals do not exhibit a high BC value, which indicates node centrality within the network. A low BC value signifies that these publications play a limited mediating role in the academic network, implying a reduced capacity to connect disparate sub-networks. Consequently, the primary contribution of these papers lies in their extensive citation rather than functioning as pivotal bridges for academic communication. This finding underscores the critical importance of enhancing communication and collaboration.

The author with the highest number of citations is Dr. Thomas Klockether (Figure 7) from the University of Bonn (Germany). He is the author of an SCA review that has been cited 29 times (1). Dr. Klockether has made outstanding contributions to this field, and his article is considered groundbreaking in SCA research.

Analysis of cited references serves as a valuable tool to reflect the knowledge foundation and identify the mainstream direction of the field. The results of our citation analysis (Table 3) indicate that the primary research focus is on utilizing MRI to investigate changes in the brain microstructure of SCA patients, aiming to optimize the diagnosis, prediction, and treatment of SCA diseases based on these findings. MRI stands as one of the most widely utilized technologies for studying SCAs. Among the research methods, structural MRI (sMRI) is particularly effective in evaluating and monitoring macroscopic morphological changes in the brains of SCAs patients (32). It quantifies the degree and pattern of atrophy in the affected areas of SCA patients (33). Voxel-based morphometry (VBM) is widely used in SCA research (34, 35) and involves a voxel-wise comparison of the local concentration of gray matter between two groups of subjects (36). Diffusion MRI is another commonly used technique to study SCAs, which provides a new perspective for quantifying microstructural damage to cerebellar structures (37). As diffusion MRI (dMRI) is a true quantitative imaging technique, its indicators can serve as potential imaging biomarkers, allowing for early detection of pathological changes and tracking and predicting subtle changes in subsequent examinations and clinical trials (38). Functional MRI (fMRI) is a relatively new imaging technique, and research using it as a keyword began in 2015. Despite its initial emergence, scholars researching SCAs have paid less attention to fMRI since 2019. This trend may indicate a shift in focus within the academic community, influenced by the increasing maturity of the technology and awareness of its limitations (39). Moreover, fMRI demands higher image quality but poses a challenge, as many patients with SCA exhibit poor cooperation during examination; however, fMRI is starting to gain traction again in 2021, likely due to improvements in the state of the art. Overall, MRI remains the best-studied surrogate biomarker candidate for polyglutamine expansion SCAs (40). In recent years, scholars have achieved significant results using various MRI research methods mentioned above, and our keyword analysis further indicates that this area is set to become one of the future research hotspots. The development of multiple MRI techniques has introduced novel research avenues for SCAs. Integrating MRI imaging with multi-omics analysis can enhance our understanding of SCAs and offer a promising approach to refining diagnostic methods and therapeutic strategies. Furthermore, the emerging role of artificial intelligence (AI) in follow-up studies may significantly influence the future trajectory of this field.

Spinocerebellar ataxias have multiple subtypes, with the most common ones being SCA1, SCA2, SCA3, SCA6, and SCA7. These subtypes collectively account for approximately 70% of dominant SCA cases (41, 42). Among them, SCA3 is the most common subtype globally (43). Correspondingly, keyword analysis also confirms that SCA3 is currently the most extensively studied subtype in this research field. SCA3 is also the most common subtype in countries such as the United States, China, and Germany (44). Notably, these countries also demonstrate high publication volumes in related research articles. In addition, keyword analysis reveals that SCA2, a subtype with a higher proportion in Italy and Spain, has also garnered considerable attention (45).

Previous neuropathological studies have shown that SCA patients exhibit varying degrees of neuronal loss in several brain regions, such as the cerebellum, brainstem, spinal cord, cerebral cortex, and basal ganglia (43, 46). The brainstem and cerebellum are the earliest, most common, and most significant regions of change in SCA patients (47–49). Correspondingly, keyword analysis shows that both regions have received considerable attention in research, and that the brainstem is the most extensively studied brain region. Due to the physiological and anatomical characteristics of the brainstem structure, fMRI and sMRI have been hindered in brainstem research for many years (50). However, advancements in imaging technology have enabled more accurate and faster brainstem imaging (51). The cerebellum, a brain region that accounts for 80% of neurons in the entire brain, has been proven to play an important role in a wide range of cognitive behaviors. New imaging techniques will be able to quantify the microstructure of the cerebellum, observe the unique tissue environment of the lobules, describe more complex cerebellar subregions, evaluate the functional status of the cerebellum, and provide a more detailed description of the occurrence and development of diseases (52).

The analysis of Burst keywords depicted that there has been a significant increase in the frequency of “feature” appearing as a keyword in recent years. This observation suggests that using MRI data as a feature to explore biomarkers of SCAs may become one of the hotspots of future research. The selection of optimal biological biomarkers can enhance the effectiveness of MRI technology in monitoring disease progression, predicting treatment efficacy, and other aspects (53).

Bibliometric analysis has certain limitations that warrant consideration. First, as bibliometric analysis is fundamentally a quantitative approach, the relationship between quantitative metrics and qualitative outcomes often remains ambiguous. Consequently, qualitative conclusions drawn from bibliometric analyses may be subject to bias. For example, relying exclusively on publication counts as an evaluation criterion may overestimate institutions engaged in frequent yet small-scale research while underestimating those producing fewer but more comprehensive studies focused on large-scale populations. Second, bibliometric research can provide only short-term projections regarding research fields. Therefore, scholars should exercise caution when making ambitious claims about research fields or their long-term implications.

## 5 Conclusion

This bibliometric analysis provides evidence for the research hotspots and frontiers in the application of MRI in SCA diseases. Our results showed that Europe, the United States, China, and Brazil have made notable and outstanding contributions to the field. With the advancement in MRI technology, sMRI and dMRI have gained widespread application in SCA research. In contrast, fMRI, a relatively new technology, still offers significant research opportunities. SCA3 has received the most attention because it affects the largest number of patients. The brainstem and cerebellum are the most extensively studied brain regions. Additionally, future research can strategically focus on exploring the application of MRI as a biomarker in SCAs.

## Data Availability

The original contributions presented in the study are included in the article/supplementary material, further inquiries can be directed to the corresponding authors.
